# 

**Published:** 1898-06-18

**Authors:** 


**Notices of Births, Deaths, and Marriages** are inserted in this column at a charge of 2s. 6d. for an announcement not exceeding four lines.

### NOTICE TO CORRESPONDENTS.

All MS., Letters, Books for Review, and other matters intended for the Editor should be addressed The Editor, "The Hospital," The Hospital Building, 28 & 29, Southampton Street, Strand, W.C.

The Editor cannot undertake to return rejected MS., even when accompanied by stamped directed envelope.

**Notice to Advertisers and Subscribers.**

All Advertisements, Orders for Copies of The Hospital, and Business Communications should be addressed to **THE MANAGER, (Not to The Editor),** The Hospital, The Hospital Building, 28 & 29, Southampton Street, Strand, London, W.C.

The Cost of Subscription, post free, is as follows:—Per week, 2½d. per quarter, 2s. 8d.; per half-year, 5s. 3d.; per annum, 10s. 6d. Foreign:—Per annum, 15s. 2d.; payable, in all cases, in advance.

**NOTICE.—Vol.XXIII. of "The Hospital" (October, 1897, to March, 1898), in handsome cloth binding, is now on sale at the Office of this Journal, price 7s. 6d. Cases for binding the Numbers contained in this Volume, 1s. 6d. Index to Vol. XXIII., 2½d., post free.**

**The Monthly Part for July will be ready on July 1. Price 6d., by post 8d.**

### BOOKS RECEIVED.

MACMILLAN AND CO.

"Allbutt's System of Medicine." Vol. V.

JOHN WRIGHT AND CO.

"Rheumatoid Arthritis; its Pathology, Morbid Anatomy, and Treatment." By Gilbert A. Bannatyne, M.D.

C. J. HEWLETT AND SONS.

"Notes on the New British Pharmacopœia, 1898."

LONGMANS, GREEN, AND CO.

"Tablets of Anatomy." By Thomas Cooke, F.R.C.S., and F. G. Hamilton Cooke. In three parts. Eleventh Edition.

J. AND A. CHURCHILL.

"Lectures on Medicine to Nurses." By Herbert E. Cuff, M.D., F.R.C.S.

SAMPSON LOW, MARSTON, AND CO.

"Twentieth Century Practice." Edited by Thomas L. Stedman, M.D. Vol. XIII.

**Periodicals, &c.**—London, St. Bartholomew's Hospital Journal, Woman's Signal, Leisure Hour, Sunday at Home, Boy's Own Paper (Summer Number), Girl's Own Paper (Supplement), Nursing Notes, Zenana, Medical Press, Critic, Sun, British and Colonial Druggist, Cantab, Monthly Register, Literary Digest, Honied Hours, Medical News, Medical Record, South African Medical Journal, Public Health Journal, London Hospital Gazette, Guy's Hospital Gazette, New Age, Westminster Review.

